# Advances on Chelation and Chelator Metal Complexes in Medicine †

**DOI:** 10.3390/ijms21072499

**Published:** 2020-04-03

**Authors:** George J. Kontoghiorghes

**Affiliations:** Department, Postgraduate Research Institute of Science, Technology, Environment and Medicine, 3 Ammochostou Street, Limassol 3021, Cyprus; kontoghiorghes.g.j@pri.ac.cy; Tel./Fax: +357-2627-2076

**Keywords:** chelators, chelating drugs, metal complexes, antioxidants, therapeutics, diagnostics, theranostics, deferiprone

Metal ions such as iron, copper and zinc are essential for life. Chelators (Chele, greek χειλή-claw of a crab) are organic molecules possessing specific ligands which have high affinity and can bind/carry metal ions and play very important roles in living systems e.g., haemoglobin, transferrin, phytochelators and microbial siderophores [1].

The acquisition of daily dietary requirements and the maintenance of a specific range of concentrations of metal ions in the tissues ensure normal daily biological, metabolic and physiological activities and bodily functions, as well as healthy living. For example, it is estimated that zinc is required for the turnover of more than 300 catalytically active zinc metalloproteins, as well as more than 2000 zinc dependent transcription factors.

Metal metabolic imbalance is associated with serious conditions such as iron deficiency anaemia, which affects about a third to a quarter of the world’s population. Similarly, many hundreds of thousands of patients are affected by iron overload due to regular red blood cell transfusions. Among the principal categories of patients with negative consequences from transfusional iron overload are chronic haematological and malignant diseases including thalassaemia, haematopoietic stem cell transplantation, sickle cell anaemia, aplastic anaemia and cancer [1]. There are also many cases of iron and copper overload as a result of increased gastrointestinal absorption, such as iron overload in idiopathic haemochromatosis or copper overload in Wilson’s disease. The progressive accumulation of iron and copper in the body could lead to major toxicities and fatalities, unless removal therapies of these metals are introduced.

In general, the accumulation of excess metal ions including iron, copper, zinc and aluminium in any organ, as well as in cellular and sub-cellular compartments is a negative prognostic factor for many diseases [1].

The therapeutic application of chelating drugs can in many of the above and also similar other cases restore metal metabolic imbalance and treat the associated diseases (Table 1). For example, the chelating drugs deferoxamine, deferiprone, deferasirox and their combinations are used daily for the treatment of iron overload in thalassaemia and the chelating drugs penicillamine and triethylenetetramine for the treatment of copper overload in Wilson’s disease. Many other chelating drugs are also used for the detoxification of other metals [2].

Metal ions are also essential for the growth and proliferation of microbes and cancer cells, which in effect makes them a target for the design of new pharmaceuticals for treating infections and cancer (Table 1) [1,3,4]. The latter two are considered to be on the top of the list of major diseases affecting humans.

There is variation in the requirements for metal ions in each cell, including different microbial cell species and cancer cell types. Selective targeting for the inhibition of the growth and proliferation of cancer cells and microbes by deprivation of essential metals can be accomplished by chelating agents with specific properties and physicochemical characteristics [3,4]. In contrast, some natural or synthetic chelating agents and medicinal drugs could have the opposite effect and increase metal delivery thus promoting the growth of cancer and microbial cells [1,4].

The treatment of cancer is a major goal for many research institutions and investigators worldwide. Anticancer therapeutic strategies involve surgery, chemotherapy, radiotherapy and their combinations. The aim of these therapeutic strategies is to reduce cancer cell proliferation and cancer growth, and, if possible, eliminate altogether the presence of cancer cells. In most cases of chemotherapy, specific anticancer drug protocols are directed to various targets in cancer cells such as those related to key metabolic protein pathways, DNA synthesis, signal transduction etc. Damage to normal cells and other toxic effects are also observed during the anticancer treatments. The risk/benefit assessments for most anticancer therapies are not optimal and new drugs and approaches are required, including the possible application of multidrug anticancer regimens aiming at different targets. Specificity in cancer cell targeting is also important and such approaches are considered to have a better therapeutic outcome. The efficacy and risk/benefit assessments of such combination approaches which may involve metal ions and chelators is in progress, with some of these at an investigational level and others in different stages of clinical development and use [1,4,5].

The therapeutic strategies for targeting cancer cell growth and proliferation by chelators may involve the modulation of protein function or other associated pathways such as the inhibition of key enzymes e.g., the iron containing ribonucleotide reductase involved in DNA synthesis, incorporation of xenobiotic metals such as Ga which could bind to nucleotide substrates and also disrupt iron and other essential metal metabolic pathways and lysosomic damage by redox active metal complexes (Table 1) [1,4,5,6]. Chelating drugs could also be used in many other anticancer approaches such as modulation of macrophage anticancer function and also in the protection from the cardiotoxicity of anticancer drugs [7,8,9]. Of particular interest in clinical oncology are platinum complexes [10]. It is estimated that about 50% of cancer patients receive platinum complex based anticancer therapy. Such platinum complexes appear to cause crosslinking of DNA, which causes the inhibition of DNA synthesis and DNA repair in cancer cells. Each of several drugs containing different platinum-based complexes appear to have selective anticancer specificity for each cancer type [10]. Similarly, the anticancer chelating drug hydroxycarbamide (hydroxyurea) which inhibits ribonucleotide reductase is also widely used in different cancer types [1,11].

Another major area affecting health and disease, which involves metal ions and chelators is free radical pathology. Increased production of free radicals and reactive oxygen species (FR/ROS) has been implicated in almost all diseases, including those involving tissue damage, as well as in cancer and aging. In almost all biological systems the catalysis of free radical production including the effects leading to oxidative stress toxicity is carried out by iron, copper and enzymes containing these metals. The inhibition of free radical toxicity by specific iron and copper chelators, supports their selective use as main or adjuvant antioxidant therapy in free radical pathology (Table 1) [1,12].

Oxidative stress toxicity due to excess FR/ROS has been shown to cause widespread molecular, sub-cellular and cellular damage, which may lead to cell apoptosis, autophagy and necrosis. In particular, cell death due to iron toxicity called “ferroptosis” has been identified in many conditions including cancer, acute kidney disease, stroke etc. Ferroptosis appears to have specific characteristics and to be different from apoptosis or other cell death pathways. In this context, the design of antioxidant therapeutic protocols which exclude the control of iron toxicity through iron chelation, appear to be limited and ambiguous in clinical settings [1,12].

A major paradox in the treatment of diseases associated with free radical pathology is the absence of antioxidant drugs in clinical practice. However, millions of people are using daily natural antioxidants for the prevention and treatment of many diseases in the context of traditional folk medicine. The rapid expansion in the use of natural antioxidants such as plant nutraceuticals, which are sold over the counter as dietary supplements for potential nutritional and therapeutic effects, as well as thousands of related reports in the medical literature suggest that many scientists and the public have in general a positive attitude in respect of such therapeutic approaches. Many natural antioxidants including plant nutraceuticals possess metal chelating properties. Some of these phytochelators such as quercetin, curcumin, 8-hydroxyquinoline and ellagic acid are the subject of ongoing clinical investigations. Similarly, several other phytochelators such as maltol, fisetin, caffeic acid, phytic acid and silibinin appear to play a role not only in antioxidant activity but other physiological processes such as metal transport and delivery and also metal detoxification [1,12,13,14,15].

Xenobiotic metal detoxification is an important area of therapeutic applications of chelating drugs, which is associated with environmental toxicology issues including those arising from heavy metals such as lead and mercury, carcinogenic metals such as cadmium and nickel and radioactive metals such as plutonium and uranium (Table 1) [1,2]. Xenobiotic metal toxicity arising from the ingestion of food and drink product contamination, as well as inhalation of polluted air is a negative prognostic factor and is thought to be associated with many diseases. In this context, millions of people are attending alternative medicine clinics worldwide for ethylenediaminetetraacetic acid (EDTA) chelation therapy, which is widely used for xenobiotic metal detoxification. Similarly, air pollution from industrial and automobile combustion waste products and especially the inhalation of specific size micro particles which contain xenobiotic metals, toxic forms of iron and also other toxins appear to be a serious health hazard and the cause of many fatalities every year. The removal of xenobiotic metals and toxic iron forms from different organs as a result of pollution is a serious challenge for personalized chelation therapy and future intoxication targeting strategies [1,2,12].

Similar intoxication chelating strategies have to be designed also in relation to toxicity concerns arising from the excessive use of diagnostic metals such as gadolinium and technetium, which are routinely used in clinical diagnosis [16].

Specific chelation targeting strategies are also required for the treatment of diseases associated with the excess accumulation of xenobiotic and essential metals in specific organs e.g., excess iron, copper, zinc and aluminium in the brain, all of which have been implicated in neurodegenerative diseases [1,17]. Such strategies should include the selection of specific chelators that can cross the blood–brain barrier such as deferiprone [18,19]. Substantial improvements have been observed in different categories of patients with neurodegenerative diseases using deferiprone including pantothenate kinase-associated neurodegeneration (PKAN) and Friedreich’s Ataxia [12,20,21,22,23,24]. Several other ongoing and planned clinical trials for the use of deferiprone and other chelating drugs in other neurodegenerative diseases with increased morbidity and mortality such as Parkinson’s and Alzheimer’s disease patients are also in progress [25,26].

Similar targeting chelation strategies could be adopted in relation to different stages of the transport, storage, utilisation or detoxification pathways of metal ions and associated proteins. For example, the interactions of chelators, metal ions and chelator metal complexes with transferrin will affect their metabolic, therapeutic, diagnostic and detoxification properties [17]. In this context, in a total of about 100 elements of the periodic table, about 40 metal ions including all essential and xenobiotic metals mentioned above e.g., iron, copper, zinc, aluminium, gallium, technetium, indium, gadolinium, nickel, cadmium, plutonium, uranium and platinum have been identified to bind or interact with plasma transferrin. The interactions are different in each case and these differences could affect associated targeting strategies including diagnostic, therapeutic and detoxification uses [17]. In particular, the ability of high doses deferiprone to remove iron and other metals from transferrin are of physiological and clinical importance [17].

The development of monitoring methods for assessing the efficacy and mode of action of chelation therapy in the context of personalised medicine is very important for future metal intoxication targeting strategies. For example, new insights in the mode of action of chelating drugs such as the monitoring of the changes in excess iron in organs during chelation therapy using the magnetic resonance imaging (MRI) T2 and T2* techniques have increased the selectivity prospects and targeting therapeutic potential of chelating drugs for different organs including the heart, brain, liver, spleen pancreas and different diseases of gross body iron overload or of focal iron toxicity such as neurodegenerative diseases [21,22,23,24,25,27,28].

A major area of the application of chelators in medicine is the diagnostic and theranostic fields (Table 1) [29,30]. The design and targeted use of different chelating agents which form complexes of variable physicochemical properties appear to affect the bodily distribution of metal radiotracers and are increasingly applied for the diagnosis of different diseases and tracking their progress. Similar targeted chelator metal complexes have been identified for the theranostic application of radiotracer metals in cancer, inflammation and other diseases [29,30].

There are major challenges ahead in relation to the new applications of chelators, chelating drugs and their metal complexes in medicine. In addition to biomedical research developments, the continuation of basic chemical research in the design of new chelators and the characterization of their physicochemical and other properties including those of their metal complexes are essential for increasing their targeting potential and therapeutic and/or diagnostic or theranostic application in medicine [1,29,30,31].

Many factors affect the mode of action of chelators, chelating drugs and their metal complexes in vitro, in vivo and in clinical conditions (Figure 1). Basic structural characteristics of chelators determine their physicochemical and other properties including their affinity for different metal ions and stability of their metal complexes, interactions with proteins, cell membrane permeability and their pharmacological and toxicological properties (Figure 1) [1,31,32].

In each case the mode of action of chelators, chelating drugs and their metal complexes will be affected by competition with other natural chelators including chelating proteins and other metal ions. Similarly, in each case the absorption, distribution, metabolism, excretion and toxicity (ADMET) profile will be different and also be affected by pharmacogenomic, metallomic, proteogenomic, metabolomic and redoxomic factors (Figure 1).

The target organs of toxicity are different for each chelator, their metabolites and chelator metal complexes. The same differences in toxicities apply to the accumulation of excess metal ions in organs. Efficacy and toxicity aspects are also affected by the underlying disease and organ function.

Recent new developments in the clinical use, application, drug interactions, metabolic and other effects of chelating drugs and their metal complexes has increased our knowledge in relation to the pivotal role of chelators and metal ions played in living systems, as well as the diagnosis and therapy of many diseases. However, many challenges lie ahead in the optimization aspects of the diagnostic and therapeutic use of chelator and chelator metal complexes.

For example, future chelator targeting processes could be designed aiming for specific organs, cells and cellular compartments, for use in the removal or delivery of essential or xenobiotic metals and for achieving better therapeutic or diagnostic or theranostic clinical results. Similarly, improved chelator platinum complexes and hydroxyurea derivatives could be designed for targeted delivery and better treatment in different types of cancer [10,11]. Investigations using deferiprone and other chelators are also in progress for targeting oxidative stress toxicity and malfunction in mitochondria, which have been implicated in cancer and other diseases [12,31,32,33,34,35,36]. New therapeutic possibilities include the design of polymeric chelators for the extraction and removal of excess essential and xenobiotic metals in the gastrointestinal tract and in environmental pollution. In contrast lipophilic chelators such as maltol could increase the absorption of iron and other metals [37,38]

Chelating drugs could generally be used as main, alternative or adjuvant therapy in many diseases, including those associated with free radical pathology, in metal detoxification, as antioxidants, anticancer and anti-infective agents, and as modulators of protein function or pathways associated with disease. In this context, improved therapeutic strategies could be developed and adopted based on combination therapies, target-specific and prodrug design methods and other aspects that could fulfil personalized medicine characteristics [31].

However, notwithstanding that such academic research initiatives may benefit millions of patients, pharmaceutical development is generally slow and based on commercial and not academic or ethical criteria [39]. In this context, the current use of available approved drugs such as deferiprone and initiatives for academic drug development in diseases with no effective treatments could speed up the pharmaceutical process and benefit related groups of patients [39].

## Figures and Tables

**Figure 1:**
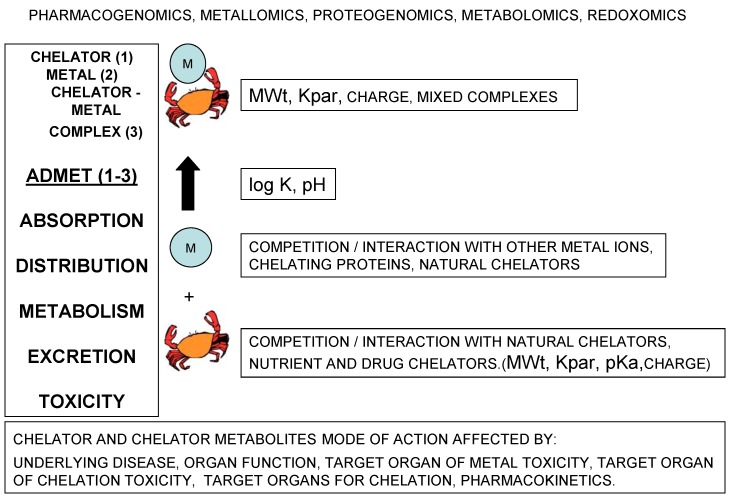
Factors affecting the efficacy and toxicity of chelators and chelator metal complexes. Many factors can influence chelating activity in biological systems including the chelator physicochemical, metabolic, pharmacolocical and toxicological properties. Some of the properties which influence the biological activity of chelators and chelator metal complexes include the equilibrium constant (log K) of metal complex formation, the molecular weight (MWt), lipid/water partition coefficient (Kpar), the charge of the chelator which depends on the pKa of ligands and the pH.

**Table 1:** Clinical applications of chelators and chelator metal complexes.

**Examples of the Clinical Applications of Chelators**
Removal of metals in diseases associated with essential metal overload All diseases related to free radical pathology Cancer Infectious diseases Neurodegenerative diseases Acute kidney disease Myocardial infarction Ageing Prevention of metal absorption in the gastrointestinal tract Xenobiotic metal decorporation originating from food and drink products, environmental pollution, the nuclear industry and weapons Decorporation of xenobiotic metals used in medical diagnosis
**Examples of the clinical applications of chelator metal complexes**
Increase metal absorption in diseases of essential metal deficiency Delivery of redox active metal complexes against cancer Delivery of xenobiotic metals to disrupt essential metal pathways in cancer Diagnostic metal delivery in diseases including inflammation and cancer Radiolabeling of cells using metal radiotracers Theranostic metal delivery in diseases
